# Association between moral distress and supporting elements of moral deliberation in nurses*

**DOI:** 10.1590/1518-8345.3990.3332

**Published:** 2020-07-15

**Authors:** Flavia Regina Souza Ramos, Laura Cavalcanti de Farias Brehmer, Graziele de Lima Dalmolin, Luciana Ramos Silveira, Dulcinéia Ghizoni Schneider, Mara Ambrosina de Oliveira Vargas

**Affiliations:** 1 Universidade Federal de Santa Catarina, Florianópolis, SC, Brazil.; 2 Universidade Federal de Santa Maria, Departamento de Enfermagem, Santa Maria, RS, Brazil.

**Keywords:** Ethics, Ethics, Nursing, Decision Making, Moral, Nursing, Stress, Psychological, Ética, Ética em Enfermagem, Tomada de Decisões, Moral, Enfermagem, Estresse Psicológico., Ética, Ética en Enfermería, Toma de Decisiones, Moral, Enfermería, Estrés Psicológico

## Abstract

**Objective:**

to identify the association between moral distress and the supporting elements of moral deliberation in Brazilian nurses.

**Method:**

a cross-sectional study conducted with Brazilian nurses working in health services at different complexity levels. The research protocol consisted of the Brazilian Scale of Moral Distress in Nurses, a sociodemographic and labor questionnaire, and a list of bases and ethical elements used for moral deliberation. For analysis, descriptive statistics, chi-square test, and Poisson regression were used.

**Results:**

1,226 nurses took part in the study. The 12 elements associated with the moral deliberation process were classified as important for nurses’ actions, especially the professional experience acquired, code of ethics/law of professional practice, and ethical and bioethical principles. The relationship of moral distress showed higher prevalence in the Beliefs, culture and values of the patient, Beliefs and personal values, and Intuition and Subjectivity elements.

**Conclusion:**

the results showed a balance between the subjective criteria of professional experience and the objective ones of deontology for moral deliberation.

## Abstract (pt)

**Objetivo:**

identificar a associação entre o distresse moral e os elementos apoiadores da deliberação moral em enfermeiros brasileiros.

**Método:**

estudo transversal realizado com enfermeiros brasileiros atuantes em serviços de saúde de diferentes níveis de complexidade. O protocolo de pesquisa constituiu-se da Escala Brasileira de Distresse Moral em Enfermeiros, questionário sociodemográfico e laboral, e uma lista de bases e elementos éticos utilizados para deliberação moral. Para análise empregou-se estatística descritiva, teste do qui-quadrado e regressão de Poisson.

**Resultados:**

participaram do estudo 1.226 enfermeiros. Os 12 elementos associados ao processo de deliberação moral foram classificados com importância para atuação dos enfermeiros, destacando-se a experiência profissional adquirida, Código de Ética/Lei do Exercício profissional, e, princípios éticos e bioéticos. A relação do distresse moral evidenciou maiores prevalências nos elementos Crenças, cultura e valores do paciente, Crenças e valores pessoais e, Intuição e Subjetividade.

**Conclusão:**

os resultados apontaram um equilíbrio entre critérios subjetivos da experiência profissional e objetivos da deontologia para a deliberação moral.

## Abstract (es)

**Objetivo:**

identificar la asociación entre el distrés moral y los elementos de apoyo de la deliberación moral en los enfermeros brasileñas.

**Método:**

estudio transversal realizado con enfermeros brasileños que trabajan en servicios de salud de diferentes niveles de complejidad. El protocolo de investigación consistió en la Escala Brasileña de Distrés Moral en Enfermeros, un cuestionario sociodemográfico y laboral, y una lista de bases y elementos éticos utilizados para la deliberación moral. Para el análisis, se utilizó estadística descriptiva, prueba de chi-cuadrado y regresión de Poisson.

**Resultados:**

1.226 enfermeros participaron en el estudio. Los 12 elementos asociados con el proceso de deliberación moral se clasificaron como importantes para el desempeño de los enfermeros, destacando la experiencia profesional adquirida, el Código de Ética/Derecho de Práctica Profesional y los principios éticos y bioéticos. La relación de distrés moral mostró una mayor prevalencia en los elementos Creencias, cultura y valores del paciente, Creencias y valores personales y, Intuición y Subjetividad.

**Conclusión:**

los resultados mostraron un equilibrio entre los criterios subjetivos de la experiencia profesional y los objetivos de ética para la deliberación moral.

## Introduction

Given the complexity of the health care system and care, nursing professionals can often face difficult situations related to structural, organizational and relational aspects among colleagues and in the professional-user relationship, during their work process. These situations involve ethical issues and require positions and deliberation, which evoke in the professionals feelings of uncertainty, discomfort and restlessness in the face of conflicts and divergences of opinions with those involved, including other professionals, patients and family members.

In this perspective, the professionals can experience moral distress, which, classified as a moral problem, occurs when they cannot conduct their action according to their judgments and personal and professional values, perceiving as inadequate their moral participation, that is, they feel powerless to act according to their conscience, either by internal or external constraints^(^ 1 ^)^.

In this sense, it can be said that moral distress is constituted from an obstruction in the process of moral deliberation, based on the individual experience and awareness about ethically appropriate conduct, that is, by interrupting the process of moral deliberation, it becomes inconclusive and fruitless because it has not reached the desired objective, producing feelings of impotence and inconvenience in the professional^(^ 2 ^)^. It is noteworthy, therefore, that the process of moral distress is inversely related to the process of moral deliberation, since the latter refers to the ability to conduct a conflicting situation in a reasonable, prudent and achievable manner, considering the values and duties involved^(^ 3 ^)^.

The moral deliberation process is a systematic and contextualized itinerary of analysis of ethical problems to find concrete solutions, among prudent alternatives. This analysis is not abstract, but considers the circumstances of the act and the foreseeable consequences. The goal of deliberation is prudent courses of action. In clinical bioethics, prudence is expressed in the ability to value what is involved in the case, with a view to reasonable decisions^(^ 4 ^)^. In the meantime, it can be said that the process of moral deliberation can constitute a tool for the ethical positioning of nurses in the face of perceived moral problems and conflicts^(^ 5 ^)^, as in situations that generate moral distress, since this has serious consequences for nursing workers, both in the personal dimension, with physical and emotional symptoms, and in the professional dimension, such as the development of burnout or even abandoning the profession^(^ 6 ^)^.

Among the factors that interfere in nurses’ moral deliberation, which may also be related to the moral deliberation process, are professional experience and practice, confidence, intuition, use of protocols, collaboration with experienced colleagues, organizational culture, education, awareness of the situation and exercising the autonomy^(^ 7 ^)^.

When considering that the moral deliberation process can be influenced by several aspects, the present study is justified by the need to identify the supporting elements of this process, that is, what may be the factors and ethical skills that favor the conduct of reflection, dialog and prudent and responsible resolution of moral problems as in the case of moral distress^(^ 8 ^)^. Thus, the objective was to identify the association between moral distress and the supporting elements of moral deliberation in Brazilian nurses.

The studies on moral distress in the field of nursing, especially in the last five years and also in Brazilian settings, have pointed out several generating situations or risk factors, strongly associated with the environment, including the structure and organization of the work process, as well as such as interpersonal relationships that are established^(^ 9 ^-^ 13 ^)^. After decades of studies, it is confirmed that the necessary and sufficient conditions to define moral suffering refer to the combination of the experience of a moral event and psychological suffering, in a direct causal relationship.

If, on the one hand, research studies emerge that show moral distress, the measurement instruments still require continuous reviews and improvements, just as it is essential to expand the discussions on the following: the impact of moral distress on the health professionals and the organizations^(^ 14 ^)^; the “nebulosity”, the limits of the concept and the elaboration of strategies to face it and the relationships with other concepts related to moral experience, such as power, moral courage, moral hazard/risk, moral sensitivity and moral deliberation in work and work and training scenarios^(^ 15 ^-^ 17 ^)^.

Thus, it is considered that identifying and discussing the dimensions related to the moral deliberation process, which translate into coping with situations of ethical conflict that generate moral distress, may contribute to the daily practice of nurses and to the advances necessary for the current stage conceptual development on the topic.

## Method

This is a cross-sectional study linked to the multicentric research developed by the Research Laboratories of three Brazilian Federal Universities.

The study participants consisted of nurses from health services of different levels of complexity throughout the Brazilian territory. To reduce bias in relation to the sample size, the minimum sample was adopted from the calculation for a finite population considering a confidence level of 95%, an error of 0.05% and a population of 451,666 nurses registered with the Federal Nursing Council in 2015, with a minimum estimated number of 385 participants. The inclusion criteria were limited to being a nurse and working for at least six months in assistance in primary health care services, medium and/or high complexity in the Brazilian scenario.

The sampling method was for convenience, in which data were collected by a virtual questionnaire (Formulários Google™) from November 2015 to May 2016 when the desired sample was met. The nurses were invited to participate by e-mail (search for registration of institutions) and through social networks using the professional denomination “nurse”. In both cases, the participants accessed the survey link, first with access to the survey information, a free and informed consent term, and after agreement, directed to the instrument. During the aforementioned collection period, e-mails were sent to numerous institutional contacts and there was an intense investment in disclosure on social networks. The availability of access to the online form was interrupted with the satisfactory number of participants.

The data collection instrument comprised the Brazilian Scale of Moral Distress in Nurses (EDME-Br)^18^ along with an item of sociodemographic and labor characterization of the participants, and a list of bases and ethical elements used for moral deliberation. The EDME-Br built up and validated in the Brazilian scenario assesses the intensity and frequency of moral distress through 57 questions on two seven-point Likert scales (from 0 = none/never to 6 = very intense/very frequent). Among the characterization variables investigated were gender, age, state of the federation, level and time of training and professional experience, type and nature of employment, and working site. The questions about moral deliberation, on the other hand, were listed based on a broad literature review on the subject of moral deliberation. From the analysis of the review studies, the main bases and elements used by nurses in the face of ethical conflicts were extracted, which were arranged in a list of 12 items in which the participants should mark on a scale from 0 to 3 the order of importance of their use in these situations.

Data was submitted to statistical analysis using the SPSS - PASW Statistic^®^ statistical software and the Predictive Analytics Software, version 23.0. Descriptive analysis was used, through distribution of relative and absolute frequency of categorical variables, and measures of position and dispersion (mean and standard deviation) of quantitative variables. For associating moral distress and bases and ethical elements, the chi-square test was used, in which the variables were dichotomized, that is, moral distress categorized as low (0 to 2.00) and moderate/high (2.001 to 6.00) considering the amplitude of the Likert scale, and bases and ethical elements in low importance (0 and 1) and high importance (2 and 3), considering the response interval. Finally, Poisson regression was used for analyzing the complete model with all the bases and ethical elements. Associations with p<0.05 were adopted as significant.

The project followed the guidelines provided by Resolution 466/2012 of the National Health Council/Ministry of Health and its complementary addendums, being approved by the Ethics Committees on Research with Human Beings (*Comitê de* Ética *em Pesquisa com Seres Humanos*, CEPSH) of all the institutions involved under opinion numbers 602.598-0 of 02/10/2014, 602.603-0 of 01/31/2014, and 511.634 of 01/17/2014.

## Results

The sample consisted of 1,226 nurses with representatives from all 26 Brazilian states and the Federal District. Among the participants, 1,148 (93.6%) were female, 512 (41.8%) were between 30 and 39 years old, 800 (65.5%) had a specialization or residency, 863 (70.8%) had only one job, 824 (67.5%) were publicly employed and 531 (43.5%) were statutory. Regarding working time, the majority, 894 (73.1%), had up to 10 years of experience. As for the nature of care, 375 (30.6%) worked in primary health care, 361 (29.4%) in secondary care and 490 (40%) in tertiary care, demonstrating an adequate representation of each level.

The bases and ethical elements used by the nurses in situations of conflict are shown in Table 1.

**Table 1:** Frequency of the bases and elements used to act ethically in the face of a conflict situation, Florianópolis, SC, Brazil, 2016

Bases and ethical elements	0* n (%)	1^†^ n (%)	2^‡^ n (%)	3^§^ n (%)	Absent n (%)	Total
1. Personal Beliefs and Values	99(8.2)	306(25.5)	392(32.6)	404(33.6)	25	1201
2. Patient’s beliefs, culture and values	37(3.1)	224(18.7)	488(40.7)	451(37.6)	26	1,200
3. Code of professional ethics /Professional practice law	14(1.2)	115(9.5)	378(31.3)	699(58.0)	20	1206
4. Professional experience gained	6(0.5)	76(6.3)	364(30.4)	751(62.7)	29	1,197
5. Intuition and subjectivity	84(7.0)	405(33.9)	443(37.1)	263(22.0)	31	1195
6. Ethical and bioethical principles	8(0.7)	107(9.0)	384(32.2)	692(58.1)	35	1191
7. Theoretical bases acquired in the training	15(1.3)	128(10.7)	390(32.5)	666(55.5)	27	1,199
8. Practices established in the service \/institution	12(1.0)	233(19.5)	497(41.7)	451(37.8)	33	1193
9. Practice agreed with the team	23(1.9)	200(16.8)	510(42.7)	460(38.6)	33	1193
10. Defense of interests and image of the profession	52(4.4)	236(19.8)	442(37.1)	462(38.8)	34	1,192
11. Defending the patient’s interests and needs	14(1.2)	133(11.1)	442(36.9)	610(50.9)	27	1,199
12. Defense of interests and image of the service/institution	69(5.7)	345(28.7)	474(39.5)	313(26.1)	25	1201

* 0 = No importance;

† 1 = Low importance;

‡ 2 = Medium/Moderate importance;

§ 3 = High importance

It is observed that all the elements listed were classified as important for ethical performance and moral deliberation by nurses, highlighting the professional experience acquired, Code of Ethics/Law of Professional Practice, and ethical and bioethical principles, in which 751 (62.7%), 699 (58.0%) and 692 (58.1%) of the respondents, respectively, attributed the greatest importance.

Table 2 shows the association between the bases and ethical elements with moral distress.

**Table 2:** Association between bases and ethical elements used for ethical performance in the face of conflicts and intensity of moral distress. Florianópolis, SC, Brazil, 2016

Bases and ethical elements		Moral distress		Total	p*
		Low n(%)	Moderate/High n(%)		
1. Personal Beliefs and Values	Low	149(37.4)	249(62.6)	1,184	0.001
	Discharge	183(23.3)	603(76.7)		
2. Patient’s beliefs, culture and values	Low	118(46.1)	138(53.9)	1,183	0.001
	Discharge	213(23.0)	714(77.0)		
3. Code of professional ethics/Professional practice law	Low	70(55.1)	57(44.9)	1,189	0.001
	Discharge	263(24.8)	799(75.2)		
4. Professional experience gained	Low	59(72.8)	22(27.2)	1180	0.001
	Discharge	271(24.7)	828(75.3)		
5. Intuition and subjectivity	Low	164(34.1)	317(65.9)	1,178	0.001
	Discharge	165(23.7)	532(76.3)		
6. Ethical and bioethical principles	Low	66(58.9)	46(41.1)	1,176	0.001
	Discharge	263(24.7)	801(75.3)		
7. Theoretical bases acquired in the training	Low	73(52.1)	67(47.9)	1182	0.001
	Discharge	258(24.8)	784(75.2)		
8. Practices established in the service/institution	Low	96(39.5)	147(60.5)	1,176	0.001
	Discharge	235(25.2)	698(74.8)		
9. Practice agreed with the team	Low	90(41.1)	129(58.9)	1,177	0.001
	Discharge	240(25.1)	718(74.9)		
10. Defense of interests and image of the profession	Low	103(36.4)	180(63.6)	1,175	0.001
	Discharge	227(25.4)	665(74.6)		
11. Defending the patient’s interests and needs	Low	82(56.9)	62(43.1)	1,182	0.001
	Discharge	249(24.0)	789(76.0)		
12. Defense of interests and image of the service/institution	Low	132(32.4)	275(67.6)	1,184	0.019
	Discharge	202(26.0)	575(74.0)		

* Chi-square test

From Table 2, it was found that the highest prevalences were found in the groups that attributed high importance to the bases and ethical elements and had moral distress at a moderate/high level. The highest prevalences of moral distress were in the groups: Patient’s beliefs, culture and values, Personal beliefs and values, and Intuition and Subjectivity.

Finally, in Table 3, the multivariate analysis of the association between moral distress and the set of bases and elements for ethical action in the face of conflicts is submitted.

**Table 3:** Regression analysis of moral distress with bases and elements for ethical performance. Florianópolis, SC, Brazil, 2016

Variables		RPb^†^	CI 95%	p	RPaj^‡^	95% CI	p
1. Personal Beliefs and Values	High	1.087	1.051-1.124	<0.001*	1.017	0.982-1.054	0.341
	Low	1			1		
2. Patient’s beliefs, culture and values	High	1.150	1.102-1.200	<0.001*	1.044	0.996-1.094	0.070
	Low	1			1		
3. Code of professional ethics/Professional practice law	Discharge	1.210	1.137-1.286	<0.001*	1.025	0.960-1.095	0.463
	Low	1					
4. Professional experience gained	Discharge	1.379	1.276-1.490	<0.001*	1.198	1.071-1.340	0.002*
	Low	1			1		
5. Intuition and subjectivity	Discharge	1.063	1.030-1.096	<0.001*	1.017	0.984-1.051	0.323
	Low	1			1		
6. Ethical and bioethical principles	Discharge	1.243	1.163-1.328	<0.001*	1.038	0.950-1.134	0.406
	Low	1			1		
7. Theoretical bases acquired in the training	Discharge	1.185	1.118-1.256	<0.001*	1.001	0.941-1.065	0.963
	Low	1			1		
8. Practices established in the service/institution	Discharge	1.089	1.045-1.135	<0.001*	0.984	0.940-1.030	0.481
	Low	1			1		
9. Practice agreed with the team	Discharge	1.101	1.054-1.150	<0.001*	1.013	0.964-1.063	0.616
	Low	1			1		
10. Defense of interests and image of the profession	Discharge	1.067	1.027-1.108	0.001*	0.972	0.933-1.012	0.171
	Low						
11. Defending the patient’s interests and needs	Discharge	1.230	1.161-1.304	<0.001*	1.114	1.035-1.199	0.004*
	Low	1			1		
12. Defense of interests and image of the service/institution	Discharge	1.038	1.005-1.073	0.023*	0.973	0.939-1.007	0.119
	Low	1			1		

* Significant value at the 0.05 level;

† RPb = Gross regression;

‡ RPaj = Adjusted regression (all variables)

It was observed from Table 3, which in the gross regression all variables remain associated with moral distress, indicating that those who attach high importance to ethical components have higher prevalences of moral distress. To the adjusted regression, where all the elements were tested together, the variables “acquired professional experience” and “defense of the interests and needs of the patients” remained associated with moral distress, with a prevalence of 19% and 11% higher, respectively, compared with those who attach low importance to ethical elements.

## Discussion

It was observed that all the supporting elements of moral deliberation were individually associated with moral distress, demonstrating a higher prevalence of moral distress in the groups that also attributed greater importance to the elements, which can be explained positively in the sense that nurses who recognize the importance of the elements, and probably use them, possibly has a moral sensitivity to perceive the moral problems and conflicts present in their daily work, applying them in an attempt to solve them.

Moral sensitivity can be understood as a personal competence that involves a contextual and intuitive aspect, configuring an essential dimension of the inter-relational aspect of nursing care in the ethical decision-making process because it allows the individual to recognize moral conflicts and people in vulnerable situations, and be aware of its consequences and implications for the others^(^ 19 ^)^.

In the combined analysis of all the elements, only two variables remained associated, which indicated a 19% and 11% higher prevalence of moral distress, that is, professional experience and the defense of patients’ interests and needs, respectively.

Professional experience and greater clinical practice can have an influence on moral deliberation in nursing because nurses with longer practice times have greater self-confidence, also relying on situations already experienced, in identifying patterns, as well as in collaboration with colleagues, to determine their decisions^(^ 7 ^)^.

It is also pointed out that, in ethical decisions, nurses use individual factors, such as personal experiences, knowledge and communication; in this case, they interconnect the experience with the greatest possibility of critical positioning, courage and ethical and moral sensitivity to conduct judgments in a sensible and judicious manner^(^ 20 ^)^. Still, when relating to moral distress, it is possible to highlight that nurses who use professional experience in the face of the deliberative processes, perceive conflicts and moral problems more clearly, suffering more.

In the same way as the element defending the interests and needs of patients, in which those who perceive conflicts seek to defend the rights of patients, exercising advocacy in nursing. Nursing advocacy has been described in different ways in the literature, both as a philosophical basis and in terms of conduct and actions to assist the patient in obtaining the necessary health care, and in protecting and defending the patient’s rights to quality care^(^ 21 ^)^.

The moral distress linked to the practice of advocacy by nurses can be due to the established barriers, such as, for example, the organizational structure and culture of health services and the power relationships present in the multi-professional team, which often neglect the knowledge of nurses and discourage them from acting according to their consciences^(^ 22 ^)^.

Still, in the individual analysis of each element that supports the moral deliberation process, high prevalence of moderate/high moral distress was identified in the elements Beliefs, culture and values of the patient, Beliefs and personal values and Intuition, subjectivity, varying between 77% and 76.3%. This result may be associated with the fact that these dimensions involve aspects of incompatibility as in the case of differences in personal and patient values and beliefs, or even in the work team, work environment and organizational norms, and may also involve uncertainty, as in the particularity of intuition and subjectivity, since it is not a formal and systematic knowledge.

Differences in values and beliefs between those involved in the same situation can be a source of conflicts for them, which in the specificity of health institutions may be due to differences in perceptions, lack of material resources, lack of personnel, budget cuts, use of technologies, power relations, lack of autonomy, vulnerability of users, among others^(^ 23 ^)^.

In the case of uncertainty, this can be understood as a moral uncertainty, which is classified as a moral problem, which occurs when the professional does not know how to determine the correct course of action ethically, but is perceived in a discomfort, in a discomfort, presenting a sense that something is not adequate when following the established course of action, choosing not to seek clarification, it may be afraid of appearing inconvenient and unreasonable^(^ 1 ^)^.

Intuition in the processes of moral deliberation stems from the unconscious perception, often based on experience, but susceptible to bias and errors, as it does not involve conscious rationality and focuses the decision mainly on emotional aspects and feelings about the patient’s condition, and not on a specific evidence. Except when intuition is triggered in the identification of similar patterns or situations, in which this recognition is seen as conscious and favors the perception of important characteristics differences within a situation, better guiding the decision^(^ 7 ^)^.

Finally, in the descriptive analysis, when punctuating the importance attributed to the use of each of the elements for moral deliberation, professional experience (62.7%), ethical and bioethical principles (58.1%) and code of ethics stood out and professional practice law (58%) with high importance.

The code of ethics and the law of professional practice are the basic legislation for nurses to act, which together with ethical and bioethical principles, provide a rationality that overcomes intuition and uncertainty, helping professionals in the face of difficulties in solving problems and moral dilemmas^(^ 24 ^)^.

The legal bases of the profession guide the actions of the professionals in health care, teaching and research, so that they may act in an ethical and safe manner, with respect for human dignity and guaranteeing quality care, as well as displaying collaboration for developing actions being responsible for exercising the autonomy. However, sometimes, there may be weaknesses in the understanding or insufficient knowledge of the code of ethics, which in the face of moral problems and dilemmas, may favor the occurrence of moral distress^(^ 25 ^)^.

The Nursing Code of Ethics can represent a guiding reference in daily practice that supports professional practice with ethical and moral awareness or, in contrast, it can exercise a mandatory relationship in arid contexts for the application of the values implied^(^ 25 ^)^. This contradiction refers to the triggering of moral distress, situations in which professionals know how to cope, but face barriers to deliberate morally.

Thus, in the face of situations that require positioning and deliberation on the part of the nurse, they often resort to the ethical principles, but also to the results and consequences of the actions, which imply reasoning and moral responsibility for deliberation. In this perspective, for prudent and correct decisions, consensus and discussion of moral problems should be used, gathering the necessary information about the case, considering the different points of view and values, to then identify the possible courses of action, negotiate them, for an adequate resolution and with respect to the involved parties^(^ 24 ^)^.

The processes of moral distress and moral deliberation are subjective constructions built in concrete environments of nursing work, therefore, interpersonal relationships and the preservation of professional values imply in strategies to overcome the ethical stagnation that causes moral distress and, thus, advance towards the deliberation^(^ 26 ^,^ 27 ^)^.

Thus, it is considered that the use of the different elements that support moral deliberation by nurses, favors a more ethical and consistent practice with personal and profession values, helping them to overcome barriers and interruptions in the deliberation process. Thus, greater qualification and ethical education are essential, which promote discussions on the moral problems and the use of tools that contribute to their resolution and exercise of the autonomy, reducing the moral distress resulting from stagnation in the face of these problematic situations.

## Conclusion

All the elements listed as supporters of moral deliberation showed an association with the moral distress. The higher the prevalence of moral distress in the nurse’s experience, the greater is the importance that they attribute to elements that can help them to face the generating situations. The results of this research showed a balance between subjective criteria of professional experience and objectives, deontology and fundamentals of ethics as a science, to resolve ethical issues.

The concept of a deliberation process is still little discussed in the nursing, as well as the solution of ethical conflicts in nursing work implies complex subjective issues. It is considered essential for the development of the profession to promote spaces for ethical/bioethical reflection on care and health work, especially for self-care and the care of the other one.
